# *De novo* Sequencing of Novel Mycoviruses From *Fusarium sambucinum*: An Attempt on Direct RNA Sequencing of Viral dsRNAs

**DOI:** 10.3389/fmicb.2021.641484

**Published:** 2021-04-13

**Authors:** Yukiyoshi Mizutani, Kazuma Uesaka, Ayane Ota, Matteo Calassanzio, Claudio Ratti, Takamasa Suzuki, Fumihiro Fujimori, Sotaro Chiba

**Affiliations:** ^1^Graduate School of Bioagricultural Sciences, Nagoya University, Nagoya, Japan; ^2^Center for Gene Research, Nagoya University, Nagoya, Japan; ^3^Department of Agricultural and Food Sciences, University of Bologna, Bologna, Italy; ^4^College of Bioscience and Biotechnology, Chubu University, Kasugai, Japan; ^5^Graduate School of Humanities and Life Sciences, Tokyo Kasei University, Itabashi, Japan

**Keywords:** mycovirus, direct RNA sequencing, *de novo* sequencing, double-stranded RNA, *Fusarium sambucinum*

## Abstract

An increasing number of viruses are continuously being found in a wide range of organisms, including fungi. Recent studies have revealed a wide viral diversity in microbes and a potential importance of these viruses in the natural environment. Although virus exploration has been accelerated by short-read, high-throughput sequencing (HTS), and viral *de novo* sequencing is still challenging because of several biological/molecular features such as micro-diversity and secondary structure of RNA genomes. This study conducted *de novo* sequencing of multiple double-stranded (ds) RNA (dsRNA) elements that were obtained from fungal viruses infecting two *Fusarium sambucinum* strains, FA1837 and FA2242, using conventional HTS and long-read direct RNA sequencing (DRS). *De novo* assembly of the read data from both technologies generated near-entire genomic sequence of the viruses, and the sequence homology search and phylogenetic analysis suggested that these represented novel species of the *Hypoviridae*, *Totiviridae*, and *Mitoviridae* families. However, the DRS-based consensus sequences contained numerous *indel* errors that differed from the HTS consensus sequences, and these errors hampered accurate open reading frame (ORF) prediction. Although with its present performance, the use of DRS is premature to determine viral genome sequences, the DRS-mediated sequencing shows great potential as a user-friendly platform for a one-shot, whole-genome sequencing of RNA viruses due to its long-reading ability and relative structure-tolerant nature.

## Introduction

Mycoviruses infect all taxonomic groups of fungi with a surprisingly wide diversity (Ghabrial et al., 2015). Most mycoviruses possess RNA genomes, including positive single-stranded (+ss) RNA, negative ss (−ss) RNA, and double-stranded (ds) RNA (dsRNA). Since Cryphonectria hypovirus 1 (CHV1) succeeded in controlling the devastating chestnut disease in Europe, mycoviruses have been attracting the attention of plant pathologists because of their biocontrol agent potential (Hong et al., 1999; Chu et al., 2002; Castro et al., 2003; Yu et al., 2010). Many mycoviruses have been identified by the short-read, high-throughput sequencing (HTS) technology, including Illumina sequencing, which expanded our knowledge of mycoviral diversity in the natural environment (Coetzee et al., 2010; Al Rwahnih et al., 2011; Bartholomäus et al., 2016; Marzano et al., 2016; Osaki et al., 2016; Zoll et al., 2018). However, the sequencing of short-read RNA viral genomes often suffers from several problems, including that it does not fully guarantee terminal sequence accuracy.

Viral RNA-dependent RNA polymerases (RdRps) generate populations of closely related viruses which are generally referred to as “quasispecies.” The error rate of nucleotide incorporation in RNA viruses is estimated to be 10^−3^ to 10^−5^ mutations per copied nucleotide, and the resultant aggregated replica makes it difficult to determine the entire genome sequence (Domingo, 2000; Vignuzzi et al., 2005). Thus, most RNA viral genome sequences reported so far can be considered consensus sequences of these populations, unless major nucleotide variations are found throughout a given viral genome. Nevertheless, RNA mycoviruses are expected to be diversified into quasispecies (Filippou et al., 2018), and such a diverse viral population should exist in a host individual or even in a single cell. Furthermore, the messenger or genomic strands of RNA viruses or both are often tightly folded to form ribonucleoproteins for viral replication, transcription, translation, or encapsidation, as well as to promote RNA functions such as those of ribozymes (Ferré-D’Amaré et al., 1998; Zhou et al., 2013). This potentially inhibits reverse transcription (RT) and polymerisation, resulting in a reduced efficiency of viral characterisation caused by a strong bias of the sequencing read depth, and fragmentation of contigs in the worst-case (Yang et al., 2012; Nasheri et al., 2017).

Recent advances in long-read sequencing technology from Oxford Nanopore Technologies may have the potential to overcome these problems. The portable sequencer (MinION) is a single-molecule DNA/RNA sequencing device that directly recognises native, individual nucleic acid molecules. It does so by reading the sequence of a single-stranded nucleic acid molecule passing through a flow cell-mounted nanopore as disruptions of current across a membrane. This enables direct sequencing of RNA molecules without the need for RNA reverse transcription or PCR amplification. The genome size of currently known RNA viruses is no more than 41 kb (Saberi et al., 2018). In this regard, previous studies demonstrated that MinION can generate RNA reads with hundreds of kb (Jenjaroenpun et al., 2018), suggesting that it has sufficient sequence coverage capability to sequence RNA viruses. Taken together, this DRS technology is a promising technique to generate a mass of head-to-tail genomic RNA sequences of unknown viruses.

RNA viruses contain dsRNA as genomic dsRNAs or as replication intermediate forms in infected cells. These viral dsRNA molecules can be easily purified by cellulose column chromatography, which is an efficient technique to separate virus-associated RNA fractions from host RNA species. In this study, we compare the performance of DRS and Illumina HiSeq in generating *de novo* viral genomic sequences from pre-set dsRNA molecules of fungal origin. The results reveal seven reconstructed viral contigs representing novel mycoviral genome sequences and raise strengths and limitations of each sequencing technology. We further discuss the potential of the use of DRS for viral RNA characterisation; it is possibly useful technical updates, as well as newly characterised mycoviruses.

## Materials and Methods

### Fungal Strains and Their Maintenance

*Fusarium boothii* strain BL13 was previously isolated from the *Fusarium* head blight-infested wheat sample in Ethiopia (Mizutani et al., 2018). This strain is infected by *F. boothii* mitovirus 1 and *F. boothii* large flexivirus 1 containing its internal deletion derivative – D-RNA. The *Fusarium sambucinum* strains FA1837 and FA2242 were screened as mycoviral reservoir strains from a fungal collection producing health-beneficial secondary metabolites in Japan (Fujimori and Chiba, unpublished data). Fungal isolates were maintained on synthetic low nutrient agar or potato dextrose agar medium at 20°C and long-term stored at 4°C. For dsRNA extraction, fungal mycelial plugs were inoculated into potato dextrose broth liquid medium and incubated for a week at 20°C without agitation.

### dsRNA Extraction

Filtrated mycelia were homogenised in the presence of liquid nitrogen, and the total RNA fractions were obtained by treatment with one round of phenol, phenol:chloroform:isoamyl alcohol (25:24:1), and chloroform:isoamyl alcohol (24:1) sequential extraction. dsRNAs were further isolated from the total RNA fractions using Cellulose Powder C (Advantec, Tokyo, Japan). To eliminate fungal chromosomal DNA and single-stranded RNA (ssRNA) species, the dsRNA fractions were further treated with RNase-free RQ1 DNase I and S1 Nuclease (Promega, Madison, WI, United States).

### Library Preparation and Sequencing

#### MinION Direct RNA Sequencing

The viral RNA library was prepared following the manufacturer’s protocol with minor modifications (SQK-RNA002, Oxford Nanopore Technologies, Oxford, United Kingdom). A total of 360 μl of dimethyl sulfoxide (DMSO) were mixed with a 40-μl solution containing 1 μg of viral dsRNA. The dsRNA was denatured into ssRNA by incubating the mix for 20 min at 65°C. The denatured viral RNA was polyadenylated using *Escherichia coli* Poly(A) Polymerase following the manufacturer’s protocol [New England Biolabs (NEB), Massachusetts, United States]. The polyadenylated RNA was then purified using Agencourt RNAClean XP kit (Beckman Coulter, California, United States) and subjected to library preparation. The RNA library was loaded on the MinION flow cell for sequencing. DRS was performed on a single R9.4/FLO-MIN106 flow cell for 2–18 h.

#### Illumina HiSeq

The cDNA library was constructed following the NEBNext Poly(A) mRNA Magnetic Isolation Module protocol (NEB). A total of 200 ng of viral dsRNA diluted in 13-μl of nuclease-free water were mixed with 4 μl of 10x first-strand cDNA synthesis buffer and 1 μl of random primer, and the mix was then incubated at 94°C for 15 min. The sample was immediately placed on ice, and 2 μl of NEBNext First Strand Synthesis Enzyme Mix was added. The sample was then incubated at 25°C for 10 min, 42°C for 15 min, and 70°C for 15 min for first-strand cDNA synthesis. Second-strand cDNA was synthesised by following the protocol for NEBNext Poly(A) mRNA Magnetic Isolation Module (Chapter 1.4–1.6). Illumina HiSeq (paired-end 85 bp) was used for sequencing, and the resulting sequence data were deposited in the DDBJ Sequence Read Archive at the DNA Data Bank of Japan (DDBJ)^1^ under the accession number DRA011337.

### Sequencing Analysis

#### MinION Direct RNA Sequencing

The raw signal DRS data were generated on MinKNOW Core version 3.6.0, and the base-called was performed using Guppy version 3.2.8 with a fast base-calling model. All nanopore reads with a quality score lower than 7 or a length lower than 300 bp were removed with Nanofilt version 2.6.0 (De Coster et al., 2018). The remaining reads were subjected to error correction using CONSENT version 2.0 with default settings (Morisse et al., 2021). *De novo* assembly was conducted using Minimap2 version 2.17-r941 and Miniasm version 0.3-r179 (Li, 2016), with the -s0.1, -c10, and -e1 command modification. The Pilon version 1.23 (Walker et al., 2014) was used to polish the raw assembly using Illumina short DNA reads. The number of reads mapping to the viral contigs was counted by Samtools version 1.7 with the flagstats command. The read coverages were visualised using SparK version 2.6.2 (Kurtenbach, 2019).

#### Illumina HiSeq

For Illumina sequencing reads, low-quality bases with a quality score lower than 30 or reads with a length lower than 20 bp were trimmed with Sickle version 1.33 (Joshi and Fass, 2011). Quality filtered reads were used for later analysis, including polishing raw assembly and assembling short reads using SPAdes version 3.13.0 (Bankevich et al., 2012) with default settings. Read coverage visualisation was conducted in the same way as with the DRS reads.

### Genomic Sequence Completion

#### Gap-Filling RT-PCR

Target cDNAs were produced using ReverTra Ace (Toyobo, Tokyo, Japan) and a gene-specific primer set from given dsRNA fragments. cDNA amplification was done with DNA polymerases, such as PrimeSTAR (Takara, Shiga, Japan) or GO-Taq (Promega, Madison, WI, United States).

#### 3'-RNA Ligase-Mediated Rapid Amplification of cDNA Ends (RACE)

The terminal sequences of dsRNAs were determined by the following method. Pre-denatured dsRNAs in DMSO (90%, 65°C) were ligated at their 3'-ends with a 5'-phosphorylated oligodeoxynucleotide, 3'-rapid amplification of cDNA ends (RACE) adaptor (5'-CAATACCTTCTGACCATGCAGTGACAGTCAGCATG-3') using T4 RNA ligase (Takara) at 16°C for 16 h. Ligated DNA-RNA strands were DMSO-denatured in the presence of the oligonucleotide 3'-RACE-1st (5'-CATGCTGACTGTCACTGCAT-3') and used as templates for cDNA synthesis. The resulting cDNA was further amplified by secondary PCR with 3'-RACE-2nd (5'-TGCATGGTCAGAAGGTATTG-3'), and gene-specific primers were designed for the desired targets.

The obtained cDNA fragments were cloned into the pGEM-Teasy (Promega, Madison, WI, United States) or pCR-Blunt cloning vectors (Thermo Fisher, Waltham, MA, United States). These were used for transformation of *E. coli* strain DH5α or TOP10 for Sanger sequencing analyses. Plasmid clones were used for BigDye sequencing (ABI, ThermoFisher, Carlsbad, CA, United States) on a 3,100-Avant sequencer (ABI/Hitachi, Foster City, CA, United States) following the manufacturer’s instructions.

### Viral dsRNAs Sequence Analysis

Sequence homology searches were performed using the Basic Local Alignment Search Tool (BLAST) algorithm provided by the National Center for Biotechnology Information,^2^ and the obtained sequence information was processed with Genetyx software (Genetyx, Tokyo, Japan). Motif searching was conducted using the online tool InterPro: protein sequence analysis and classification^3^ (Finn et al., 2017).

### Phylogenetic Analysis

Multiple amino acid (aa) alignment was constructed using MAFFT version 7^4^ (Katoh et al., 2019). MEGA-X (Kumar et al., 2018) was used to draw and visualize Maximum-likelihood (ML) phylogenetic trees, and bootstrapping analyses were conducted for blanch supporting estimation.

## Results

### DRS and Illumina Sequencing of Viral dsRNA

**Figure 1:**
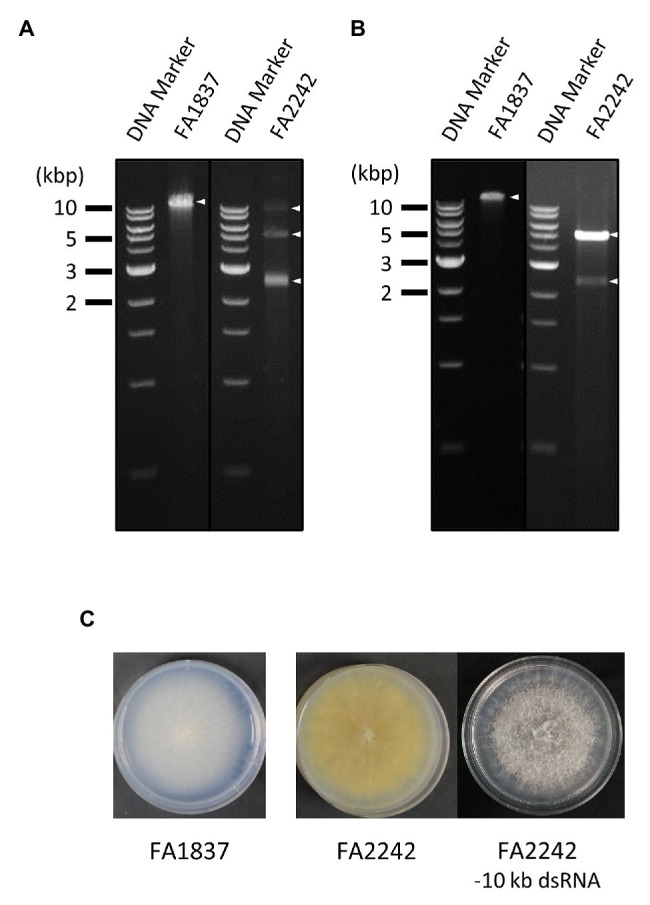
Agarose gel electrophoresis of double-stranded (ds) RNA (dsRNA) samples used for direct RNA sequencing (DRS) and Illumina HiSeq, and colony morphology of the fungal strains. **(A,B)** Agarose gel electrophoresis of dsRNA fraction used for DRS **(A)** and Illumina HiSeq **(B)** extracted from the *Fusarium sambucinum* strains FA1837 and FA2242. The 1 kb DNA ladder (NEB) and dsRNA samples were loaded on the left and right lanes, respectively. The triangle indicates the position of a viral dsRNA band. **(C)** Colony morphologies of FA1837, FA2242, and an FA2242 variant lacking the 10 kb dsRNA. The 10 kbp dsRNA element may cause reduced colony growth and pigmentation and increased ariel hyphae.

*Fusarium sambucinum* strains FA1837 and FA2242 were isolated from a Japanese *Fusarium* collection as dsRNA-positive isolates (unpublished data). Agarose gel electrophoresis revealed that FA1837 accumulated a single dsRNA band with more than 10 kbp, and FA2242 carried multiple dsRNA bands ranging from 2.5 to over 10 kbp (Figure 1A). Based on three trial DRS runs using a known viral dsRNA sample from *F. boothii* BL13 (Supplementary Material), we observed that the option of RNA library preparation that generated the longest sequence reads was the one using reverse transcription to unfold the RNA structure without the dsRNA gel extraction step to eliminate non-viral RNAs. Thus, this library preparation scheme was applied for *de novo* sequencing of FA1837 and FA2242 dsRNA samples. As summarised in Table 1, sufficient reads and bases were recovered from the DSR runs of FA1837 (44,947 reads/35,385,181 bases) and FA2242 (346,720 reads/227,987,879 bases) after trimming of short- or low-quality reads. The maximum and average read lengths were 10,571 and 787.3 nucleotides (nt), respectively, for the FA1837 RNA sample, and 7,664 and 657.6 nt, respectively, for the FA2242 RNA sample.

**Table 1:** Sequencing statistics of DRS and Illumina HiSeq.

Method	Sample	Run time (h)		Number of reads	Total bases	Read length			Viral reads (%)
						Minimum	Average	Maximum	
DRS	FA1837	2	Raw data	75,139	40,831,406	1.0	543.0	10,571	
			Trimmed (>q7, >300 bp)	44,947	35,385,181	300.0	787.3	10,571	79.25
	FA2242	12	Raw data	530,510	263,876,004	1.0	497.4	7,664	
			Trimmed (>q7, >300 bp)	346,720	227,987,879	300.0	657.6	7,664	73.34
Illumina	FA1837		Raw data	230,836	17,774,372	77.0	77.0	77	
			Trimmed (>q30, >20 bp)	80,978	5,391,822	20.0	66.6	77	71.08
	FA2242		Raw data	787,482	60,636,114	77.0	77.0	77	
			Trimmed (>q30, >20 bp)	588,732	39,558,717	20.0	67.2	77	94.65

For comparative analyses, the conventional HTS method with Illumina HiSeq was performed using dsRNA extracted from the same *F. sambucinum* strains. After read trimming, this method resulted in 80,978 reads with 5,391,822 bases for FA1837 and 588,732 reads with 39,558,717 bases for FA2242 (Table 1). However, the largest dsRNA fragments (>10 kbp) in FA2242 were accidentally lost in the dsRNA fraction that was used for Illumina HiSeq, probably because of the long-term, low-temperature storage or successive subculture on potato dextrose agar media (Figure 1B). Of note, the FA2242 strain without the >10 kbp dsRNA developed larger aerial hyphae but the colonies were smaller in size and less yellow in colour as compared to the strain containing this segment (Figure 1C). This phenotypic alteration may be associated with 10 kbp fragment, or with distinct dsRNA proportions of 5.0 and 2.5 kbp fragments, since the amounts of these dsRNAs were apparently different in the FA2242 strain with and without the >10 kb fragments (Figures 1A,B).

### *De novo* Assembly and BLAST Analysis

The *de novo* assembly of the FA1837 DRS reads generated one large contig (Contig 1; 12,721 nt). BLASTX analysis of this sequence revealed that it had the highest identity (25.83%) with the polyprotein encoded by a member of the family *Hypoviridae*, namely *Fusarium graminearum* hypovirus 1 (FgHV1; Table 2). Six contigs were generated by *de novo* assembly using DRS reads of FA2242 and were subjected to BLASTX analysis. All six sequences showed homologies to RdRps of known mycoviruses. These contigs were named Contigs 2–7 in decreasing order of size. The largest contig (Contig 2) was 10,380 nt long and had the highest identity (29.36%) to the polyprotein encoded by *Fusarium oxysporum* dianthi hypovirus 2. Contig 3 was 5,119 nt long and showed similarity to the RdRp and coat protein (CP) of Botrytis cinerea victorivirus 1 (BcVV1; 47.42 and 46.15% identities; Table 2). The remaining four contigs (Contigs 4–7) had a similar length of approximately 2.5 kb (2,791, 2,560, 2,468, and 2,292 nt, respectively). The deduced aa sequences of the encoded proteins showed the highest similarity to the reported mitoviral RdRps, with 30.04–78.86% identities (Table 2).

**Table 2:** Results of the BLASTX analysis of contigs generated from DRS and Illumina reads.

Sample	Name	Contig no.	Length	Average coverage	Closest relative virus (BLASTX)	Accession	*E* value	Identity (%)
***DRS***								
FA1837	FsamHV1	Contig1	12,721	3,012.0	polyprotein (*Fusarium graminearum* hypovirus 1)	AZT88611.1	6.0E−11	25.83
FA2242	FsamHV2	Contig2	10,380	3,901.0	polyprotein (*Fusarium oxysporum* dianthi hypovirus 2)	QHI00074.1	9.0E−69	29.36
	FsamVV1	Contig3	5,119	10,061.3	RNA-dependent RNA polymerase (Botrytis cinerea victorivirus 1)	QBA69889.1	3.0E−37	47.42
					coat protein (Botrytis cinerea victorivirus 1)	QBA69888.1	1.0E−12	46.15
	FsamMV1	Contig4	2,791	1,940.4	RNA-dependent RNA polymerase (Plasmopara viticola lesion associated mitovirus 24)	QIR30247.1	2.0E−52	37.08
	FsamMV2	-	-	26,562.2^*^	-	-	-	-
	FsamMV3	Contig5	2,560	1,424.7	RNA-dependent RNA polymerase (Soybean leaf-associated mitovirus 4)	ALM62249.1	0.0E+00	78.86
	FsamMV4	Contig6	2,468	21,908.7	RNA-dependent RNA polymerase (Plasmopara viticola lesion associated mitovirus 26)	QIR30249.1	5.0E−40	30.04
	FsamMV5	Contig7	2,292	884.3	RNA-dependent RNA polymerase (Plasmopara viticola lesion associated mitovirus 46)	QIR30269.1	1.0E−55	35.19
***Illumina***								
FA1837	FsamHV1	Contig8	13,109	305.2	hypothetical protein FgHV1gp2 (Fusarium graminearum hypovirus 1)	YP_009011065	0.0E+00	39.06
FA2242	FsamVV1	Contig9	2,725	44.8	putative RNA dependent RNA polymerase (Magnaporthe oryzae virus 1)	YP_122352.1	0.0E+00	42.57
		Contig10	2,358	34.2	coat protein (Botrytis cinerea victorivirus 1)	QBA69888	6.0E−165	48.04
	FsamMV1	Contig11	2,650	838.8	RNA-dependent RNA polymerase (Soybean leaf-associated mitovirus 5)	ALM62240	0.0E+00	82.12
	FsamMV2	Contig12	2,629	10,629.7	RNA-dependent RNA polymerase (Botryosphaeria dothidea mitovirus 1)	QMU24933	6.0E−130	41.64
	FsamMV3	Contig13	2,577	793.5	RNA-dependent RNA polymerase (Soybean leaf-associated mitovirus 4)	ALM62249	0.0E+00	78.86
	FsamMV4	Contig14	2,473	2,237.6	RNA-dependent RNA polymerase (Plasmopara viticola lesion associated mitovirus 26)	QIR30249	0.0E+00	59.11
	FsamMV5	Contig15	2,418	435.6	RNA-dependent RNA polymerase (Plasmopara viticola lesion associated mitovirus 46)	QIR30269	0.0E+00	73.52

* The average coverage of DRS reads mapped to FsamMV2 contig generated from Illumina reads.

The *de novo* assembly of the FA1837 Illumina reads generated 23 contigs. Only the largest contig (Contig 8; 13,109 nt) showed a significant similarity to a viral factor in the BLASTX search, which is a hypothetical protein encoded by FgHV1 with 39.06% aa identity (Table 2). Additionally, the *de novo* assembly using the FA2242 Illumina short reads generated 82 contigs in total. The BLASTX analyses of these contigs revealed six putative aa sequences showing significant homology to viral RdRp sequences (Contigs 9 and 11–15) and one putative CP sequence (Contig 10; Table 2). Contigs 9 and 10 were 2,725 and 2,358 nt long, respectively. The deduced aa sequences of the encoded proteins showed the highest identities with the putative RdRp of Magnaporthe oryzae virus 1 (MoV1, 42.57%) and the putative CP of BcVV1 (48.04%). The other five sequences (Contigs 11–15) had 2,650, 2,629, 2,577, 2,473, and 2,418 nt, respectively. The Contigs 11–15 coding protein sequences showed the highest similarities to RdRps of the following mitoviruses: soybean leaf-associated mitovirus 5 (SlaMV5; 82.12% identity), Botryosphaeria dothidea mitovirus 1 (BdMV1, 41.64%), SlaMV4 (78.86%), Plasmopara viticola lesion-associated mitovirus 26 (PVaMito26, 59.11%), and PVaMito46 (73.52%), respectively (Table 2). Consistent with the absence of the largest dsRNA in the FA2242 Illumina sample (Figure 1B), no hypovirus-derived reads were detected in Illumina HiSeq data.

**Figure 2:**
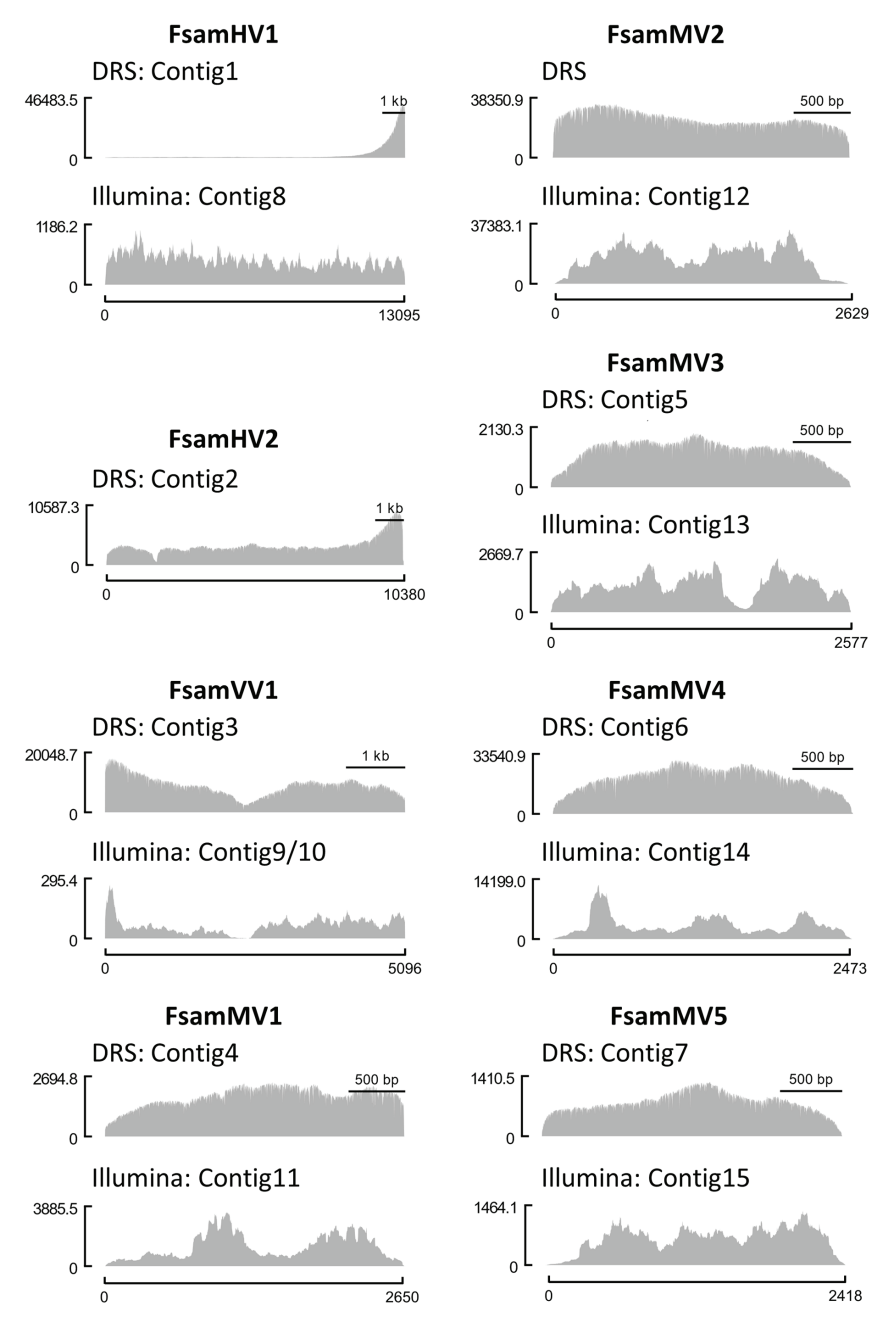
Comparison of coverage uniformity between DRS and Illumina HiSeq. Genomic coverages of each viral contig from DRS (upper graph) and Illumina HiSeq (lower graph) visualized as histograms. The Illumina contigs of *F. sambucinum* hypovirus 1 (FsamHV1) and *F. sambucinum* mitoviruses (FsamMVs) and the DRS contig polished with Illumina reads of *F. sambucinum* victorivirus 1 (FsamVV1) were used as reference sequences for read mapping. The *x*-axis indicates the nucleotide position of a viral genome, and the *y*-axis indicates the coverage depth at each nucleotide position.

We then mapped the DRS and Illumina reads on viral contigs and visualised the read coverages along the contigs (Figure 2). Altogether, the read coverages of DRS were more uniform than those of Illumina HiSeq. However, regions with extremely biased read coverage were observed on two viral contigs: DRS reads were significantly enriched at the 3′-end of the hypovirus-like sequence (Contigs 1 and 2), and both DRS and Illumina reads were significantly unrepresented in the central part of the victorivirus-like sequence of FA2242 (Contigs 3, 9, and 10).

### Sequencing Analysis

**Figure 3:**
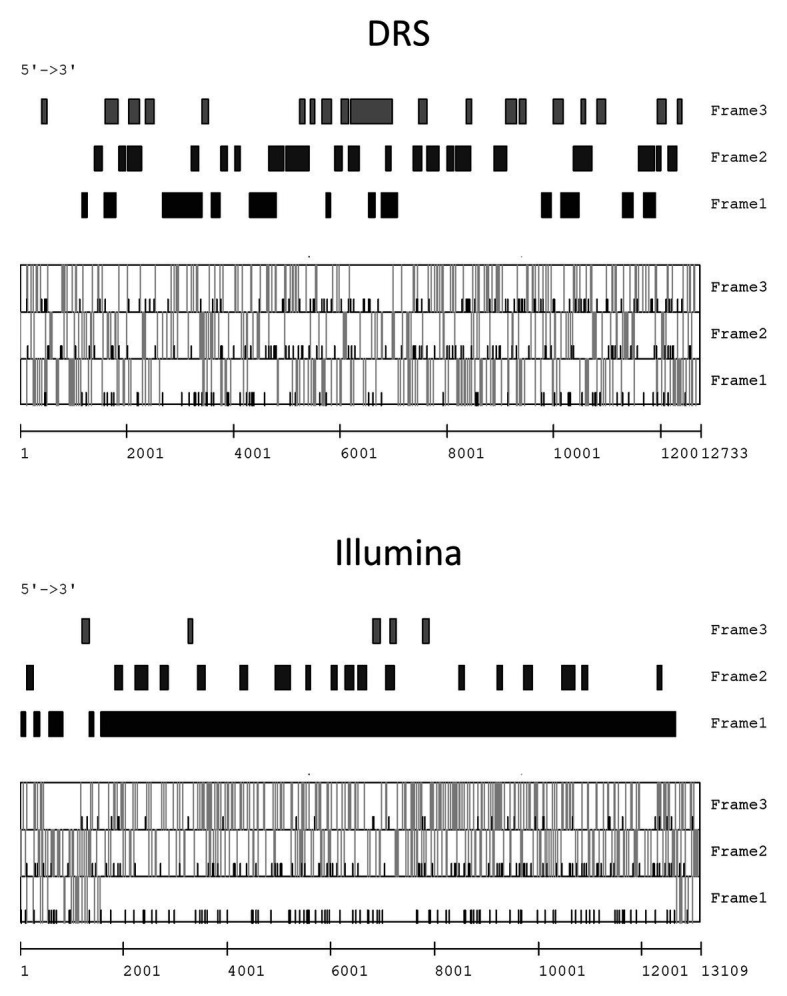
Open reading frame (ORF) prediction of FsamHV1 contigs derived from DRS and Illumina reads. Prediction of the coding regions larger than 100 bp on the sense strand of FsamHV1 generated from DRS and Illumina reads **(upper panels)**. Prediction of the initiation codon (AUG) and the termination codons (UAA, UAG, and UGA) positions on each reading frame **(lower panels)**. Black bars indicate positions of initiation codons, and gray bars indicate those of termination codons in the lower panels.

The prediction of open reading frames (ORFs) based on the contigs generated from DRS and Illumina reads was performed. To predict the mitovirus sequences, the mitochondrial codon table was applied, whereas for the other sequences, the standard codon table was used. Well-established ORFs were predicted from all Illumina contigs, whereas no obvious ORFs were predicted in the DRS contigs. For example, in the FA1837 hypovirus, one large ORF was predicted from one Illumina contig (Contig 8); however, many small ORFs interrupted by stop codons were predicted from the corresponding DRS contig (Contig 1; Figure 3). Therefore, the DRS technology appeared to be unsuitable to determine the whole viral genome sequence at this stage. Consequently, the following studies used Illumina contigs as basis for the determination of viral genomic RNA sequences. In particular, the terminal and gap regions of a hypovirus-like sequence from FA1837 and victorivirus-like sequences from FA2242 were further verified by RACE and RT-PCR.

#### Genome Organization and Phylogenetic Placement of the Putative Hypovirus Detected in FA1837

**Figure 4:**
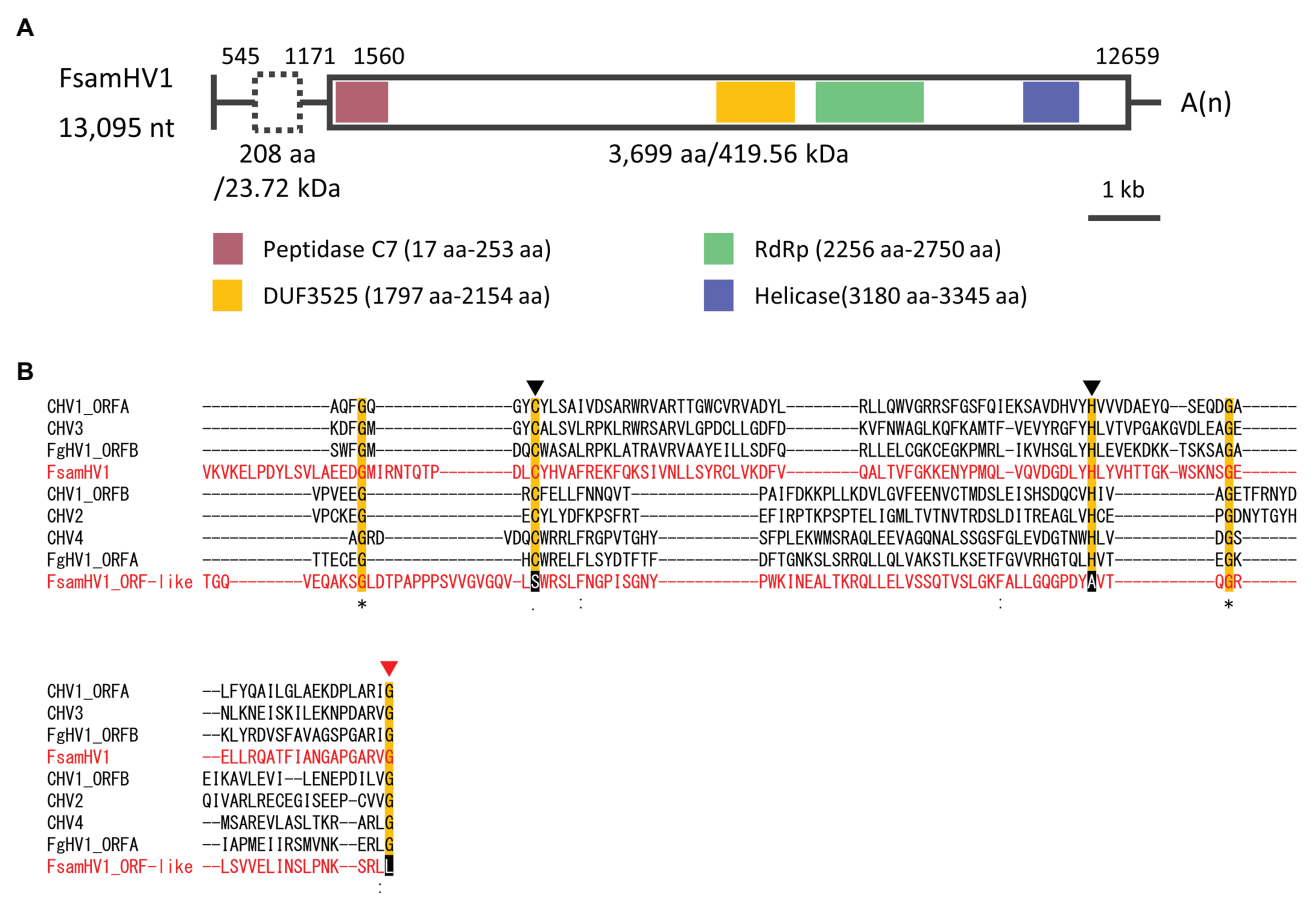
Genomic properties of FsamHV1. **(A)** Schematic representation of the complete genome of FsamHV. Open box with solid line, predicted ORF; open box with dashed line, putative ORF with GUG as a start codon; red box, conserved peptidase domain; yellow box, DUF3525 domain with unknown function; green box, conserved RNA-dependent RNA polymerase (RdRp) domain; and blue box, conserved helicase domain. **(B)** Amino acid (aa) sequence alignment of the region corresponding to the peptidase domain. Amino acid residues, which are required for the autoproteolytic activity, are indicated by black triangles, and an amino acid residue of the putative cleavage site is indicated by a red triangle.

The genomic characterisation of the viral contig-coding strand in FA1837 is shown in Figure 4A. The refined viral sequence was 13,095 nt long (excluding the poly-A sequences at the 3'-end) and its G + C content was 47.8% (accession number LC596823). The genome consisted of 1,559 nt 5'-untranslated region (UTR), 11,100 nt ORF and 436 nt 3'-UTR (excluding the poly-A). The large ORF putatively encodes a polypeptide of 3,699 aa with a calculated molecular mass of 419.56 kDa. As commonly seen among the polyproteins encoded by the *Hypoviridae* family members, three functional domains – a peptidase C7 domain, an RNA replicase superfamily (RdRp domain), and a helicase ATP-binding domain – were predicted to be present in this order from the N- to the C-terminus of this putative polyprotein. Besides those domains, a conserved domain with an unknown function named DUF3525 was also predicted upstream of the RdRp domain. Alphahypoviruses were classically distinguished from betahypoviruses by the number of ORFs they contain (Yaegashi et al., 2012). However, recently reported viruses have one large ORF [e.g., *Alternaria alternata* hypovirus 1 (AaHV1) and Wuhan insect virus 14 (WhIV14)], whereas the classical members have a small ORF encoding a protease in addition to a large polyprotein ORF (e.g., CHV1, CHV2, and FgHV1; Suzuki et al., 2018).

The ORF prediction detected one large ORF on the FA1837 hypovirus genome. Moreover, a region with 708 nt that is not interrupted by stop codons was present upstream of this ORF (Figures 3, 4A). Of note, only the last 150 nt of this region were predicted to be translated when the AUG triplet is used as a start codon. It is known that seven non-AUG codons function as start codons in *Neurospora crassa* (Wei et al., 2013). For example, when the GUG was applied to this case, the region was predicted to encode a 208 aa polypeptide with a molecular mass of 23.72 kDa. The BLASTP analysis revealed that most of this potential polypeptide sequence had a similarity to the hypothetical protein encoded by the ORF A of FgHV1 (identity: 31.94%, query cover: 91%; Supplementary Table S1), although no significant nt sequence similarities were found. The putative polypeptide of FgHV1 ORF A was predicted to contain a peptidase domain, which shows a similarity to the peptidase domain of the polyprotein encoded by CHV4 (Wang et al., 2013). A multiple alignment of the predicted peptidase domains of the FA1837 hypoviral large polyprotein and of the potential small protein suggested that the latter was inactive. In fact, the key aa residues (Cys^167^ and His^219^) required for autoproteolytic activity and the putative cleavage site (Gly^253^) were conserved only in the protease domain of the large polyprotein (Figure 4B; Koonin et al., 1991; Yuan and Hillman, 2001). This observation leads to speculations about the reason for the presence of such an unusually long 5'-UTR in the genome of this hypovirus.

**Figure 5:**
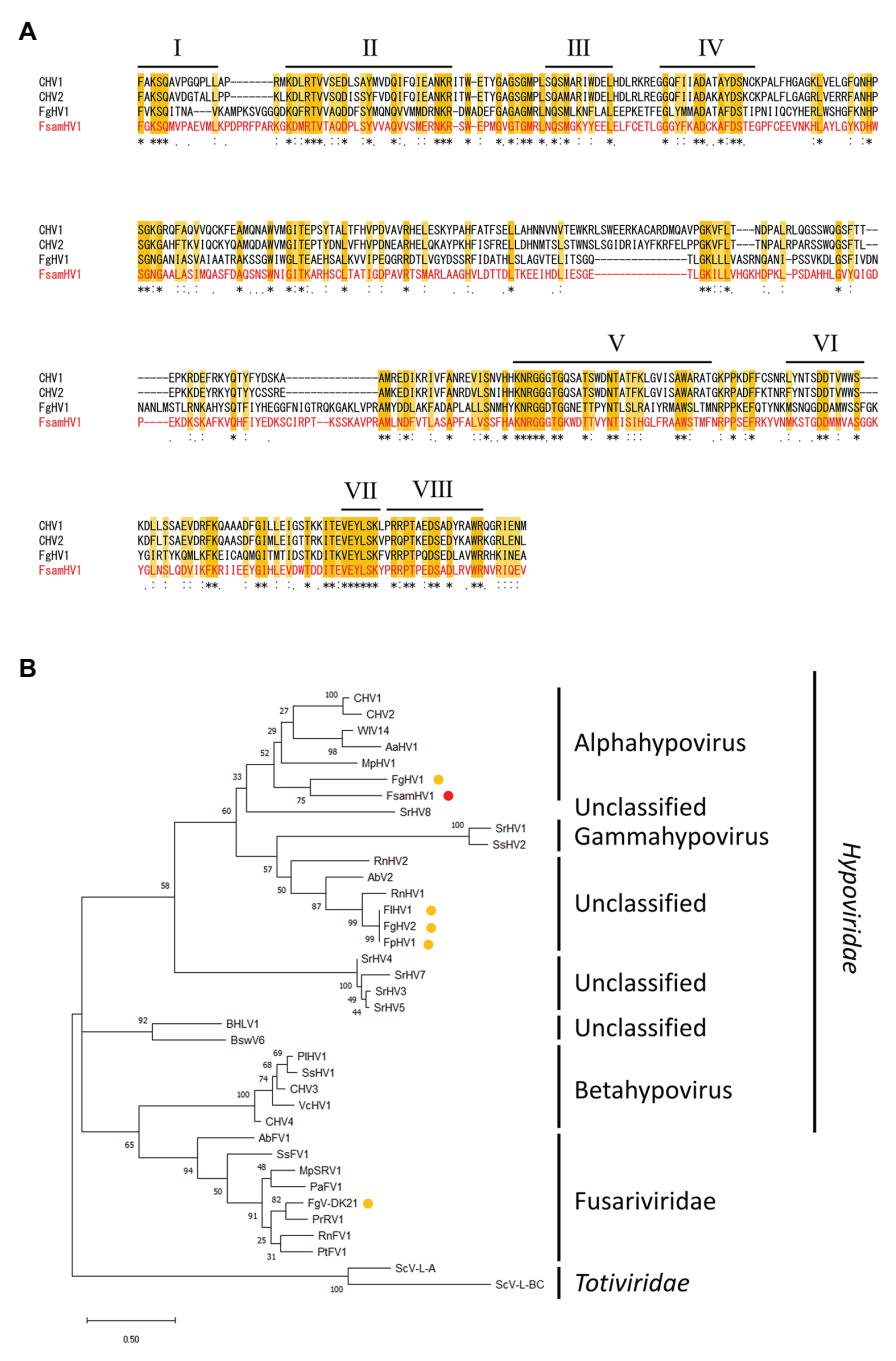
Multiple alignment and phylogenetic placement based on the replicase sequence of FsamHV1. **(A)** Amino acid sequence alignment of the region corresponding to the RdRp domain. The position of eight core RdRp motifs and conserved residues (Koonin et al., 1991) are highlighted. **(B)** Maximum-likelihood (ML) phylogenetic tree based on the multiple alignment of amino acid sequences of conserved RdRp domain predicted on the putative replicases of FsamHV1 and on other definitive and tentative members of the families *Hypoiviridae* and Fusariviridae. Two members of the *Totiviridae* family were used as the outgroup. A red dot indicates FsamHV1, and yellow dots indicate hypoviruses and fusariviruses found in the *Fusarium* species. Bootstrap values obtained with 100 replicates are indicated on branches. Branch length corresponds to the genetic distance; the scale bar at the lower left corresponds to a genetic distance of 0.50. A list of full virus names and accession numbers used in this analysis is summarized in Supplementary Table S2.

Next, the aa sequence alignment of the putative RdRp domain of the FA1837 hypovirus and other members of the *Hypoviridae* family was constructed. While alphahypoviruses possessed a consensus SDD tripeptide in RdRp motif VI, FA1837 hypovirus and FgHV1 contain a GDD tripeptide, suggesting that they have a close evolutionary relationship (Figure 5A). An ML phylogenetic analysis based on the putative RdRp domain of the FA1837 hypovirus, other members of the *Hypoviridae* family, members of the phylogenetically closest relative Fusariviridae family, and two members of the *Totiviridae* family (outgroup) was performed (Figure 5B). The result showed that the FA1837 hypovirus was part of a sub-group with FgHV1 within a cluster of alphahypoviruses, consistent with the result of the BLAST analysis and alignment of the RdRp domain. The FA1837 virus is considered to belong to a new species of the proposed genus *Alphahypovirus* in the family *Hypoviridae*, although clear species demarcation criteria have not been specified by the International Committee on Taxonomy of Viruses (ICTV; Suzuki et al., 2018). Hence, this FA1837 hypovirus is tentatively named *F. sambucinum* hypovirus 1 (FsamHV1).

In addition, although the determination of hypo-like viral sequence (Contig 2) in FA2242 was not completed and its taxonomic placement was uncertain, the FA2242 hypovirus was tentatively named *F. sambucinum* hypovirus 2 (FsamHV2) based on the above-mentioned homology search. Moreover, the loss of >10 kbp dsRNA in FA2242 (Figures 1A,B) was coincident with no detection of FsamHV2-associated reads in the Illumina-based HTS (Table 2), thus the dsRNA segment was highly expected to be a replicative form of FsamHV2. In this connection, the phenotypic changes in FA2242 variants might associate with FsamHV2 infection (Figure 1C).

#### Genome Organisation and Phylogenetic Placement of the Putative Victorivirus Detected in FA2242

**Figure 6:**
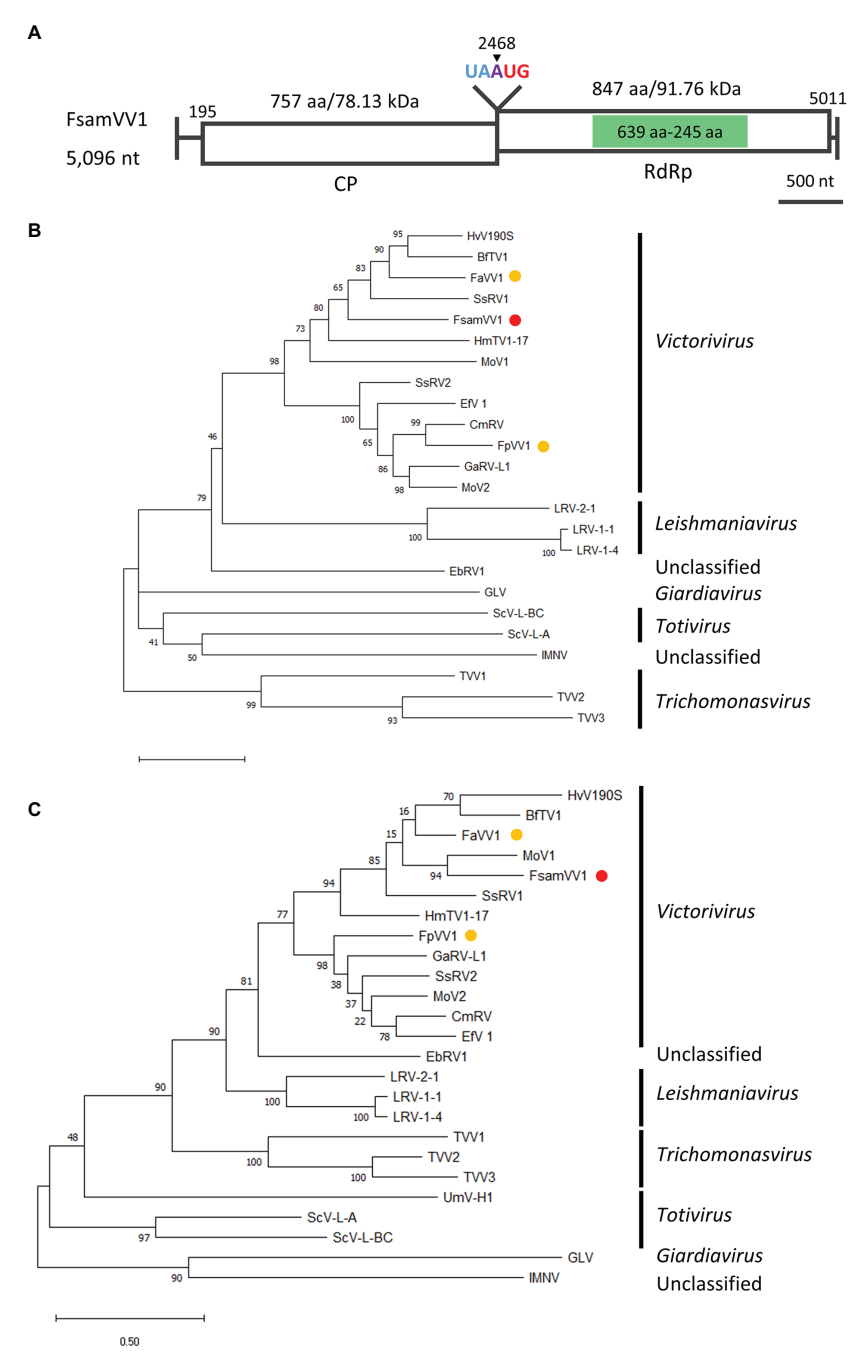
Genome organization and phylogenetic placement of FsamVV1. **(A)** Schematic representation of the complete genome of FsamVV1. White boxes indicate putative ORFs, and green boxes indicate the conserved RdRp domain predicted by InterProScan. **(B,C)** ML phylogenetic tree of CP **(B)** and RdRp **(C)** of FsamVV1 and other approved and predicted members of the *Totiviridae* family. A red dot indicates FsamVV1, and yellow dots indicate victoriviruses isolated from *Fusarium* species. Bootstrap values obtained with 100 replicates are indicated on branches. Branch length corresponds to the genetic distance; the scale bar at the lower left corresponds to a genetic distance of 0.50 in **(B)** and 0.20 in **(C)**. A list of full virus names and accession numbers used in this analysis can be found in Supplementary Table S2.

Refining victorivirus Contigs 9 and 10 in the FA2242 strain revealed a full-length dsRNA genome sequence with 5,069 nt (accession number LC596824). The genome consists of 194 nt 5'-UTR, 2,274 nt CP-ORF, 2,544 nt RdRp-ORF, and 58 nt 3'-UTR (Figure 6A), and its G + C content is 66.82%. Furthermore, a “UAAUG” pentamer was found between the two ORFs and a putative H-type pseudoknot structure upstream of the pentamer was predicted, which are typical molecular attributes of victorivirus that may facilitate the coupled translation termination and re-initiation of the two ORFs (Figure 6A; Supplementary Figure S1). The CP- and RdRp-ORFs putatively encode polypeptides with 757 and 847 aa and a calculated molecular mass of 78.12 kDa and 91.75 kDa, respectively. BLASTP analysis of the putative CP and RdRp revealed that the highest sequence identity to the corresponding proteins of *Victorivirus* genus members were the CP of BcVV3 (46.83%) and the RdRp of MoV1 (42.69%). The CPs of victoriviruses generally have an Alanine-Glycine-Proline-rich region on their C-terminal region and, indeed, this feature was also detected on the CP of the FA2242 victorivirus, especially on the most downstream 50 aa (Supplementary Figure S2). Moreover, a multiple alignment between the RdRp domain encoded by the FA2242 virus and selected members of the *Victorivirus* genus revealed eight conserved motifs, including the core GDD tripeptide (Supplementary Figure S3). The family *Totiviridae* consists of five genera: *Totivirus*, *Victorivirus*, *Trichomonasvirus*, *Giardiavirus*, and *Leishmaniavirus* (Wickner et al., 2011). Among these, only the *Totivirus* and *Victorivirus* accommodate fungal viruses. By performing the ML phylogenetic analyses based on the multiple alignments of CPs and RdRps, we observed a clear taxonomic placement of the FA2242 virus in the genus *Victorivirus* and family *Totiviridae*, which is distantly related to other victoriviruses of the *Fusarium* species (Figures 6B,C). Based on the species demarcation criteria proposed by the ICTV, viruses belonging to the *Victorivirus* genus are considered different species if the aa sequence identity of either the CP or RdRp proteins is lower than 60% (Wickner et al., 2011). Because both gene products of the FA2242 victorivirus have lower sequence identities than this threshold, this virus was named *F. sambucinum* victorivirus 1 (FsamVV1) and represents a potential novel species in the *Victorivirus* genus.

#### Genome Organization and Phylogenetic Placement of the Putative Mitoviruses Detected in FA2242

**Figure 7:**
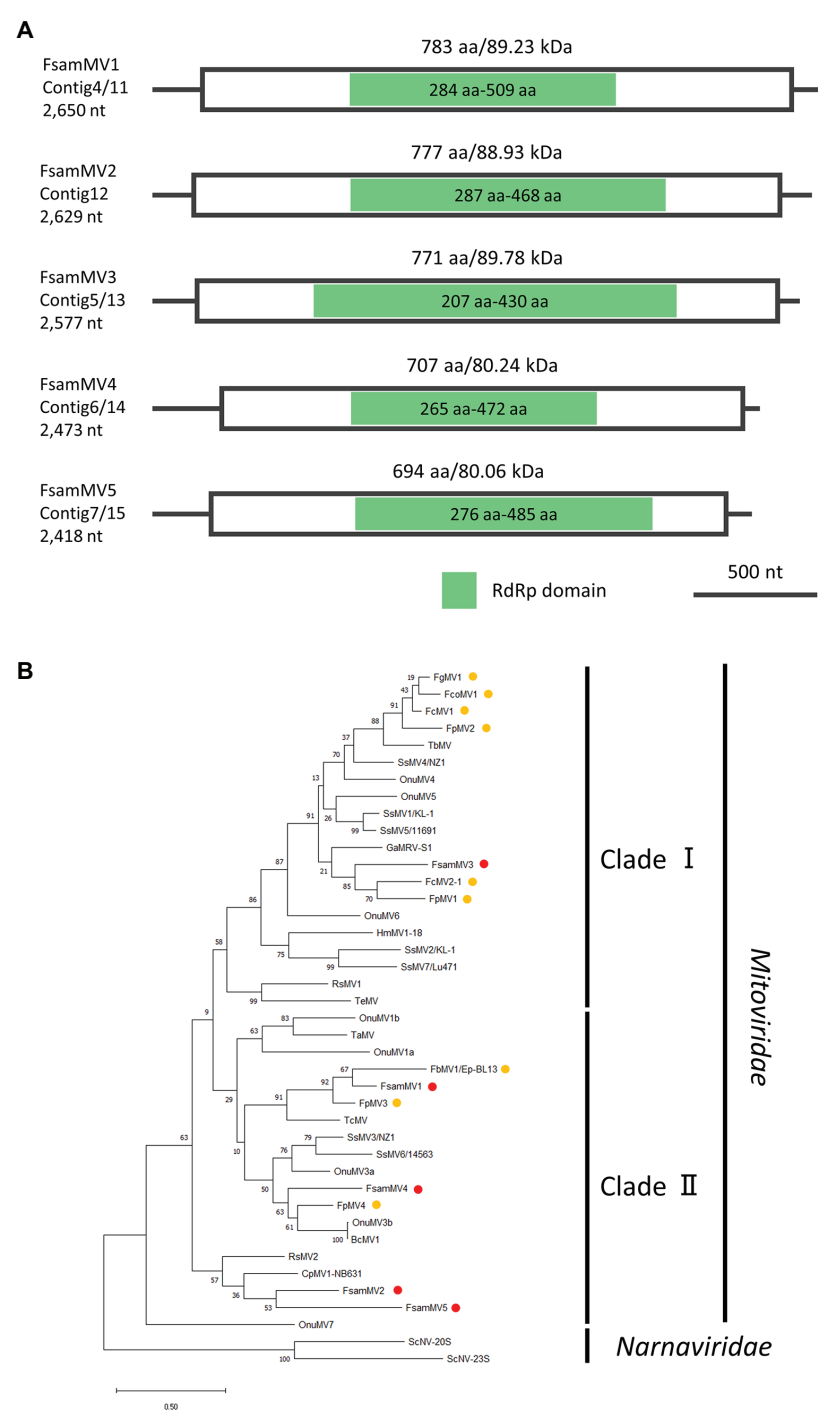
Genome organization and phylogenetic placement of FsamMVs. **(A)** Schematic representations of the contigs of FsamMVs. White boxes indicate putative ORFs and green boxes indicate the conserved RdRp domain predicted by InterProScan. **(B)** An ML phylogenetic tree based on the multiple alignment of amino acid sequences of the conserved RdRp domain predicted on the putative replicases of five FsamMVs and other members of the *Mitovirus* genus. Two members of the *Narnavirus* genus were used as the outgroup. Red dots indicate FsamMVs, and yellow dots indicate mitoviruses of the *Fusarium* species. Bootstrap values obtained with 100 replicates are indicated on branches. Branch length corresponds to the genetic distance; the scale bar at the lower left corresponds to a genetic distance of 0.20. A list of accession numbers of the sequences and full virus names are summarized in Supplementary Table S2.

Taking in consideration, the genomic structures of nearly full-genome sequences of mitovirus-like contigs (Contigs 11–15, Figure 7A) and the phylogenetic analysis of deduced aa sequences (Figure 7B), FA2242 viruses were named as follows: Contig 11, *F. sambucinum* mitovirus 1 (FsamMV1); Contig 12, FsamMV2; Contig 13, FsamMV3; Contig 14, FsamMV4; and Contig 15, FsamMV5 (accession numbers LC596825–LC596829). As commonly seen in the genomes of mitoviruses, the FsamMV contigs showed a relatively low G + C content (28.09–38.96%). Furthermore, the FsamMV replicases have an aa length and molecular mass of 783 aa and 89.12 kDa (FsamMV1), 777 aa and 88.85 kDa (FsamMV2), 771 aa and 89.84 kDa (FsamMV3), 707 aa and 79.93 kDa (FsamMV4), and 694 aa and 79.68 kDa (FsamMV5). A multiple alignment of RdRp domain encoded by the FsamMVs and selected *Mitovirus* members revealed that the replicases of all FsamMVs have six conserved motifs with the core GDD tripeptide (Supplementary Figure S4). The ML phylogenetic tree further showed that FsamMV3 was clustered into clade I and that the other FsamMVs were clustered into clade II of the *Mitovirus* genus. According to the species demarcation criteria for the genus *Mitovirus* of the ICTV, viruses are considered a different species if their RdRp sequence identities are <40%, while different strains of the same species share RdRp sequence identities >90% (Hillman and Esteban, 2011). Considering that the highest aa sequence identities of the RdRps in these FsamMVs were between the two values (41.64–82.12%, Table 2), the FsamMVs characterised in this study may represent strains of five novel species within the genus *Mitovirus*, the family *Mitoviridae*.

## Discussion

In this study, we conducted *de novo* dsRNAs sequencing of novel mycoviruses with long-read (DRS) and short-read (Illumina HiSeq) sequencing methods. While both techniques succeeded in reconstructing nearly full-length viral genomes, the DRS did not retrieve any viral contig sequence with sufficient accuracy that is good enough for the ORF prediction. Based on the short-read sequencing, seven mycoviruses have been characterised, i.e., one hypovirus and one victorivirus (representing type strains of novel species of the *Alphahypovirus* and *Victorivirus* genera, respectively) and five mitoviruses, of which the complete genome sequences were determined for FsamHV1 and FsamVV1. To the best of our knowledge, this is the first report of mycoviruses identified in *F. sambucinum*. The comparison of the sequencing efficiency between Illumina HiSeq and DRS revealed that the DRS technology is still on a developing curve to achieve the determination of viral genomic sequences by *de novo* assembly. Here, we discuss the performance of the DRS technology in this study and the potential solutions for the future application of DRS to dsRNA sequence determination.

### Sequence Accuracy of DRS

Since the sequence accuracy of the DRS reads is not high enough compared with that of the DNA ion tolerance sequencing (MinION DNA, error rate <5%), virologists have only used DRS to detect viruses in clinical samples, to analyse the RNA modification of viral genomes, and to investigate complex viral genomic structures (Quick et al., 2016; Keller et al., 2018; Kim et al., 2019; Lewandowski et al., 2019; Wongsurawat et al., 2019; Depledge and Wilson, 2020). This method has so far not been used successfully for sequence determination in virus research. Previously, high consensus sequence accuracy (up to 98.97% to the reference) was achieved in the DRS-based sequencing of the zika virus with a high read-depth by using a virus-specific adapter but not oligo-dT adapter and a reference-based assembler (Keller et al., 2018). However, those consensus sequences still contained errors, which suggest that DRS cannot be applicable for sequence determination based on *de novo* assembly with its present performance. In this study, we attempted to overcome these sequence errors by enriching the read depth with a selective template (purified viral dsRNAs) that is exclusive of host-associated RNA species and by refining contigs with available bioinformatics tools (see Supplementary Material). Even though the resultant error rates were still considerably high, the effort improved sequencing accuracy up to 92.88–96.74% (Supplementary Table S3). Nevertheless, as the DNA sequencing accuracy of the same device has dramatically improved in the last 5 years (less than 60% to approximately 95% raw read accuracy; Goodwin et al., 2016; Kono and Arakawa, 2019), it is anticipated that this DRS method experiences a further increase in accuracy in the near future.

### Characteristics of DRS Output When Using dsRNA Templates

The average length of DRS reads obtained in this study (672.5 nt) was significantly shorter than those reported in other studies. The plausible reason is the use of different RNA templates: we used dsRNA molecules in this study, whereas previous studies relied on ssRNAs (Quick et al., 2016; Keller et al., 2018; Kim et al., 2019; Lewandowski et al., 2019; Wongsurawat et al., 2019; Depledge and Wilson, 2020). Because the Oxford Nanopore DRS method is designed for mRNA sequencing, here the viral dsRNAs were required to be physically modified into a suitable form [ssRNA with poly(A)] by heat-denaturation in the presence of DMSO. It should be noted that the majority of the DRS reads were internal sequences of dsRNAs that did not cover the extremities of the target, suggesting that polyadenylation has mainly occurred in internal dsRNAs regions (Figure 2). Hence, the dsRNA might have been damaged by vigorous vortex mixing during cellulose column chromatographic purification, creating nicks, where poly(A) tailing could occur *in vitro*. It is also possible that the library construction was inhibited by insufficient dsRNA denaturation or re-hybridization of denatured RNAs or both. Considering the fact that a severe degradation of dsRNAs was not observed after the denaturation step (data not shown), the latter reasons seem more likely.

RNA viruses often carry poly(A) tails at the 3'-end. This modification is present in the dsRNA, either as a dsRNA genome (such as in partitiviruses) or as a replication intermediate of an ssRNA genome (such as in hypoviruses). We here found a strong bias of reads at the 3'-end of FsamHV1, and to some extent, of FsamHV2 (Figure 2). This is expected because the 3'-poly(A) is subjected to the tether attachment of the flow cell, and thus naturally polyadenylated RNAs should dominantly access to the pores. Additionally, the low efficiency of the polyadenylation reaction may cause a lower sequencing coverage in most of the genomic regions than in the 3'-end, in which the coverage of the 5'-end (3'-terminus of the complementary strand) was not as deep as in the 3'-region. This suggests that the coverage uniformity and sequencing efficiency of DRS vary depending on the presence of poly(A) tails and on the efficiency of the polyadenylation reaction. Overall, an improved RNA library construction may provide key advancements for viral dsRNA DRS if it: (1) assures proper dsRNA denaturation with long-term maintenance as ssRNA; (2) achieves effective polyadenylation or adaptor ligation of the target 3'-ends; and (3) avoids potential nick insertions in dsRNA to enrich head-to-tail reads.

Finally, the UTRs of RNA viruses generally carry important cis-elements that form secondary/tertiary structures or distant base-pairing or both. Comparing the 5'- and 3'-ends of the FsamHV1 and FsamVV1 complete genomes (experimentally determined) with those of the DRS contigs, we observed that all of the termini sequences of DRS and Illumina contigs were longer than those of the complete genomes, suggesting a high variation of terminal sequences in the viral genomes (Supplementary Figure S5). Because we did not find nucleotide positions that showed a clear match to the termini of the genome – which were inferred by a drastic coverage change described in a previous report (Urayama et al., 2016) – neither the DRS nor the Illumina methods can be considered applicable for terminal sequence determination.

### Significance of Enriched Viral RNAs as a Template of DRS

Contamination of host-derived reads has been a major problem for DRS application in virus sequencing. Therefore, the use of purified viral RNA elements as DRS templates poses a great advantage to specifically recover viral reads. In this regard, viral genomic RNA purified from virions resulted in a high yield of viral DRS reads by limiting the contaminated host read to ~60% (Kim et al., 2019). Likewise, the ratios of viral reads in this study were significantly higher (79.25% in FA1837 and 73.34% in FA2242; see Table 1) than those in other studies (less than 41%; Kim et al., 2019; Lewandowski et al., 2019; Wongsurawat et al., 2019). This difference was caused by the use of enzymatic degradation of contaminated host ssRNAs in the dsRNA fraction. Although the degradation products were still retained in the dsRNA fraction and represented a certain portion of reads by DRS, excluding these contaminants by gel purification of dsRNA bands is not recommended due to the reduced yield of DRS reads (see Supplementary Material and Supplementary Table S4). The dsRNA fraction can be obtained more easily if compared with viral genomic RNA purification from virions. However, the dsRNA enrichment procedure requires a certain amount of starting biological material, which depends on the levels of dsRNA present in the organisms being studied. Of note, a dsRNA purification method using a dsRNA binding protein was already established for starting materials with limited availability and may be useful for dsRNA-based viral DRS analyses (Atsumi et al., 2015).

### Potential Advantages and a Caution of Long-Read DRS During Viral dsRNA Assessment

One of the benefits of long-read sequencing is to have non-fragmented contigs that enable the skipping of gap-filling PCR analyses. In this regard, ion torrent sequencing such as Pacific Biosciences Sequencing may be a feasible option to have long-read sequences (Boldogkői et al., 2019; Myers et al., 2020). However, the method may still have a concern for the dependency on cDNA library construction. Looking at our case in the *de novo* assembly of FsamVV1 (FA2242) with Illumina short reads, the obtained contigs were divided at the boundary of two ORFs (Figure 2; Table 2; contigs 9 and 10). The members of the family *Totiviridae*, which possess dsRNA genomes with partially overlapping ORFs encoding CP and RdRp, often contain a GC-rich region that hampers complete sequencing. The downstream RdRp-ORF is translated by −1 frameshifting as a CP-RdRp fusion (*Totivirus*), or by coupled translation termination-re-initiation as two independent proteins (*Victorivirus*; Wickner et al., 2011; Ghabrial and Nibert, 2009; Li et al., 2011). The latter mechanism is a non-canonical translation that is governed by pseudoknot structures. As the fragmentation of victorivirus and totivirus contigs at the corresponding region has been seen in virome analysis using short-read deep sequencing (Marzano and Domier, 2016; Urayama et al., 2016), it is plausible to assume that this genomic region prevents library constructions (Nasheri et al., 2017; Jamal et al., 2019). This was clear in the short-read sequencing of FsamVV1 (Figure 2), where the Sanger sequencing of an RT-PCR product from this region was not easily achieved. On the other hand, the *de novo* assembly using DRS reads was able to reconstruct concatenated contigs of FsamVV1 (Figure 2; Table 2). In this case, the sequence of RT-PCR fragments was almost identical to the DRS consensus, although its read depth was not as high as in other regions. These results suggest that DRS is more amenable to sequence regions where Illumina cannot be applied and can be used for connecting divided RNA contigs.

Unexpectedly, we were not able to reconstruct the FsamMV2 genome by *de novo* assembly using DRS reads, but were able to do so from the Illumina reads. Recently, a study demonstrated that DRS data are capable of differentiating viral strains that are 20–40% divergent (Tan et al., 2019). Considering that the nucleotide sequence similarities to other FsamMVs were only up to 50% and enough DRS reads (26562.1 × average coverage) were mapped to FsamMV2 (Table 2; Supplementary Table S5), neither merging of FsamMV2 reads to other FsamMVs contigs nor the limitation of read amount can explain this result. This observation was confirmed to occur in many different assembly conditions, and thus it is recommended to subject non-reconstructed reads for a second round of assembly.

### Unique Genomic Feature of the Hypovirus FsamHV1

The members of the *Hypoviridae* family possess non-segmented +ssRNA genomes that are 9–13 kb in length. The prototypic members of alphahypovirus (CHV1 and CHV2) contain two ORFs (A and B): the ORF A of CHV1 encodes the p29 cysteine protease, which functions as an RNA silencing suppressor, and also p40, which is cleaved by p29-autocatalitic activity; the downstream ORF B encodes the replication-associated polyprotein, which corresponds to the protein conserved in all hypoviruses (Suzuki et al., 2018). However, recently reported alphahypoviruses (AaHV1 and WhIV14) were shown to possess a single ORF (Li et al., 2019), and FsamHV1 showed a single-protein coding capacity in this study, suggesting that the bicistronic nature should not represent a part of genus demarcation criteria. Interestingly, FsamHV1 was found to have an unusually longer 5'-UTR (1,559 nt) than the majority of hypoviruses (~0.5 kb), but exhibited a potential coding capacity for a 208 aa protein using the GUG start codon in this region. Of seven non-AUG start codons used in *N. crassa*, the GUG codon is functional at a 7% efficiency level of that of the AUG codon (Wei et al., 2013). The usage of non-AUG start codons increases in starvation conditions in budding yeast (Ingolia et al., 2009), indicating that the translation regulation of the ORF responds to the host metabolic conditions. Many viruses have *cis*-acting genomic regions upstream of their ORFs, known as internal ribosomal entry site (IRES), which promote the initiation of protein synthesis by cap-independent non-canonical mechanisms (Walsh and Mohr, 2011). Recently, IRES elements were found in the genome of three hypoviruses (CHV1, CHV2, and CHV3; Chiba et al., 2018). Moreover, the IRES elements of some viruses such as members of the *Dicistroviridae* family enable the translation initiation from a non-AUG codon (Touriol et al., 2003; Jan, 2006). Concerning the presence of multiple stem-loop structures predicted on the upstream region of the putative coding 5'-UTR region of FsamHV1 (Supplementary Figure S6), a non-AUG initiating IRES may exist at an upstream position of this putative ORF and express a protein. Thus, investigating the IRES activity of this RNA region is of great interest.

## Data Availability Statement

The datasets presented in this study can be found in online repositories. The names of the repository/repositories and accession number(s) can be found at: NGS data (DRA011337) and accessions LC596823–C596830.

## Author Contributions

CR, FF, and SC: designed the research and obtain research funds. FF: provided the research materials. YM, AO, MC, and TS: executed the experiments. YM, KU, MC, FF, and SC: performed the data and bioinformatic analyses. YM, KU, and SC: wrote the manuscript. FF and SC: edited the manuscript. All authors contributed to the article and approved the submitted version.

### Conflict of Interest

The authors declare that the research was conducted in the absence of any commercial or financial relationships that could be construed as a potential conflict of interest.

## Footnotes

^1^http://www.ddbj.nig.ac.jp/

^2^http://www.ncbi.nlm.nih.gov/

^3^https://www.ebi.ac.uk/interpro/

^4^https://mafft.cbrc.jp/alignment/software/
