# Supplementary material for: De novo Sequencing of Novel Mycoviruses From Fusarium sambucinum: An Attempt on Direct RNA Sequencing of Viral dsRNAs
Source: Front Microbiol. 2021 Apr 13;12:641484. doi: 10.3389/fmicb.2021.641484 (PMC8076516; doi:10.3389/fmicb.2021.641484)
Supplement: Supplementary file 2 [file Data_Sheet_1.doc]

Supplementary Material

# Supplementary Data

**The optimization of library preparation protocol for direct RNA sequencing (DRS) by using FbLFV1 and FbMV1 infecting *F.boothii* srain BL13**

To optimize sample preparation and library construction protocol, we re-sequenced viral genomes of Fusarium boothii large flexivirus 1 (FbLFV1) (accession No. [LC425115](https://www.ncbi.nlm.nih.gov/nuccore/LC425115)), FbLFV1 D-RNA (accession No. [LC425116](https://www.ncbi.nlm.nih.gov/nuccore/LC425116)), and Fusarium boothii mitovirus 1 (FbMV1) (accession No. [LC425112](https://www.ncbi.nlm.nih.gov/nuccore/LC425112)) whose genomic sequence were previously reported (Mizutani et al., 2018). Prior to library preparation, we extracted viral dsRNA from mycelia of the host strain *F. boothii* BL13, and heat-denatured it into ssRNA at 65℃ for 20 min, under the presence of 90% DMSO. Viral ssRNA was poly(A) tailed to their 3' ends as described in the previous reports (Kim et al., 2019; Wongsurawat et al., 2019). The reverse transcription step is recommended in the manufacturer’s protocol (SQK-RNA002) to keep RNA molecules unfolded from secondary and tertiary structures, that contribute to accurate and efficient sequencing. The sequencing library was prepared basically following the manufacturer’s protocol, but we tried with or without the reverse transcription step that can be skipped.

For the 1^st^ run, we tried a protocol mentioned in (Wongsurawat et al. 2019); the reverse transcription step was omitted from the manufacturer’s protocol. After an 18-hour run, we obtained 10,800 reads in total with the largest read length of 4,880 nt. Almost half of the reads (47.6%) were discarded after quality trimming with a quality score lower than 7 or a length lower than 300 bases. 76% of the remaining reads were mapped to the viral reference sequences. Interestingly, a large portion of the reads (61%) were mapped to FbMV1 genome (Supplementary Table S4), despite its band intensity of agarose gel electrophoresis was comparable to or weaker than those of FbLFV1 and FbLFV1 D-RNA, and only about 26% of viral reads were mapped to FbLFV1 or its D-RNA.

In the 2^nd^ run, the sequencing library was prepared following the manufacturer’s protocol without modifications. As a result, an increased number of reads (136,580 reads) was obtained from the same amount of input RNA with increased read quality. In addition, the maximum length of the viral read was doubled (10,712 nt) compared to that of the 1^st^ run.

In the 3^rd^ run, we conducted agarose gel purification of dsRNA before denaturation and poly(A) tailing, to eliminate potentially fragmented viral RNA molecules and contaminated ssRNAs, in order to enrich longer viral reads. The resulting data was significantly improved in terms of quality, average length, and the ratio of viral reads (Supplementary Table S4). Notably, gel purification enriched reads derived from FbMV1 specifically, while the ratio of FbLFV1 and D-RNA reads was significantly reduced. The dsRNA concentration after gel purification was roughly checked by agarose gel electrophoresis, but a significant reduction of dsRNA concentrations of FbLFV1 or its D-RNA was not observed (data not shown). According to the result, the gel purification step might not be suitable for any kind of virus dsRNA preparations, at least not applicable to sequencing FbLFV1 genome because the step led to a significant reduction of reads from specific virus elements and of detection sensitivity.

Based on the results from the three trial runs, it was concluded that the second experimental flow was a suitable condition for *de novo* sequencing of dsRNA samples of FA1837 and FA2242.

# Supplementary Figures


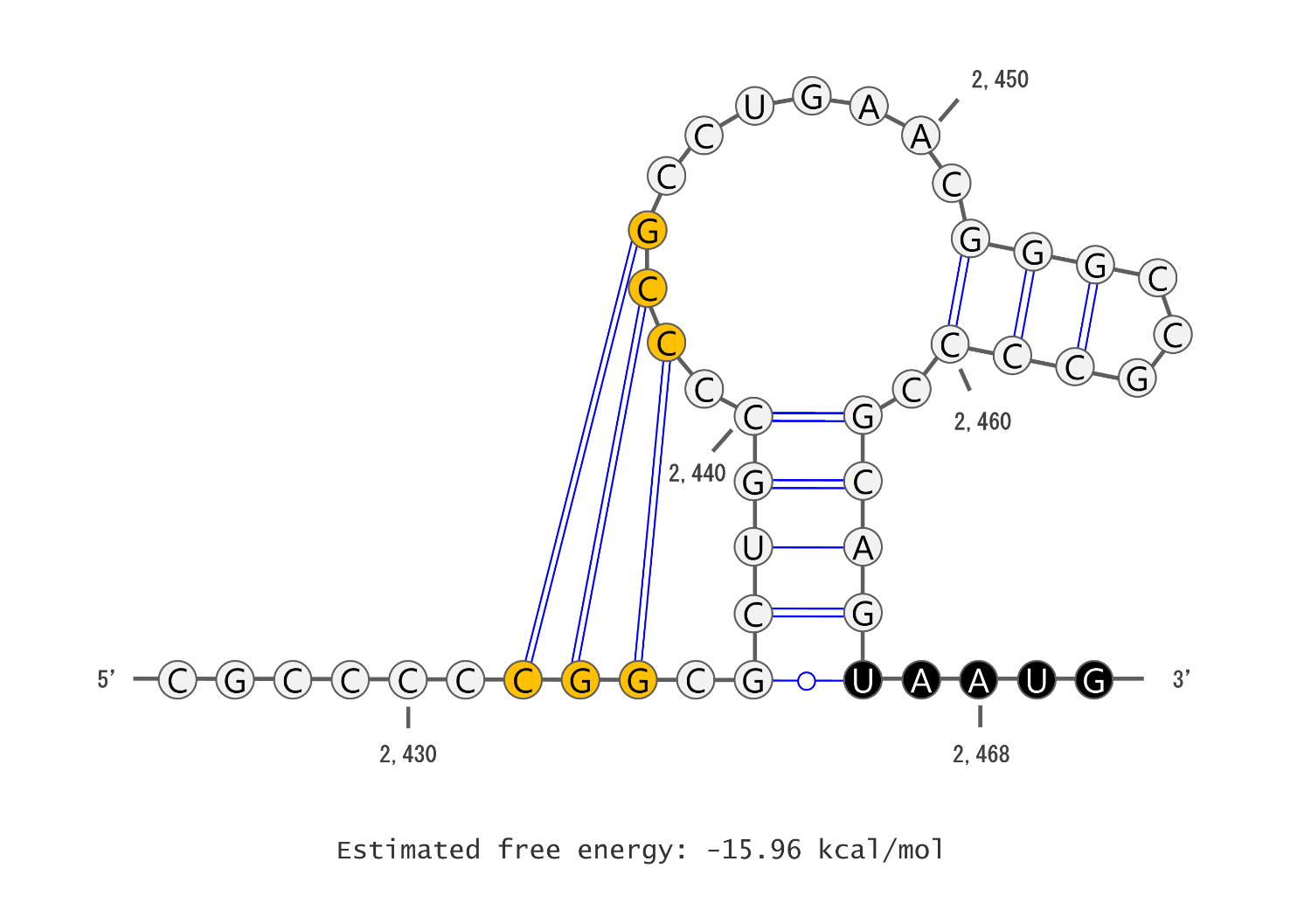


**Supplementary Figure S1.** Schematic representation of the putative secondary structure of a portion of FsamVV1 genomic RNA. The 45 nt upstream of the start codon of RdRp-ORF (nucleotide position: 2,426 nt – 2,470 nt) was subjected to the folding prediction using DotKnot (<https://dotknot.csse.uwa.edu.au/>) and visualized by VARNA version 3-93. The overlapped stop and start codons (A UAAUG pentamer) are highlighted by black.


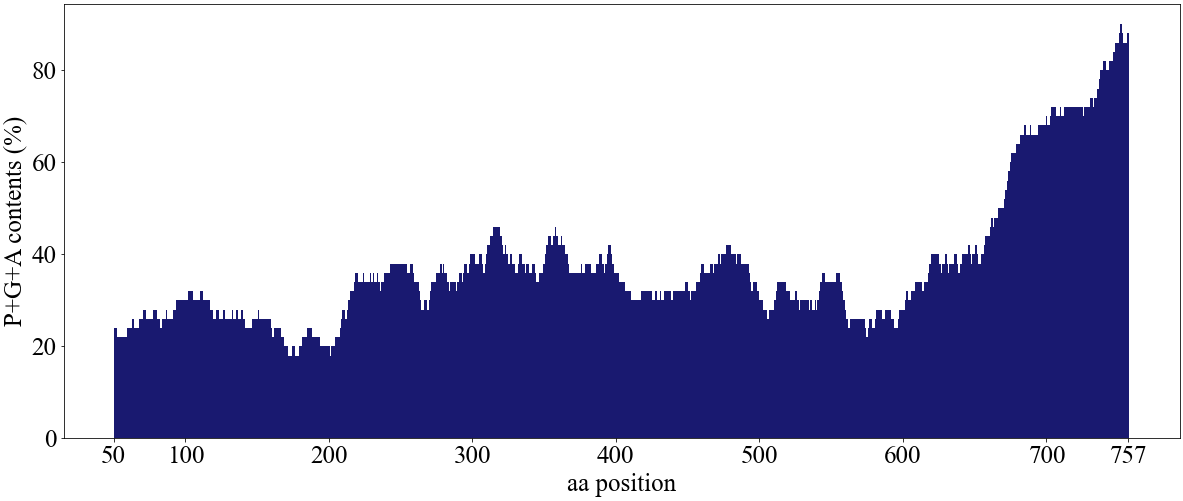


**Supplementary Figure S2.** A histogram of Ala/Gly/Pro contents of the putative CP encoded by FsamVV1 genome. The x-axis indicates the amino acid position of the CP and the y-axis indicates the ratio of Ala/Gly/Pro contents per 50 amino acids before each position.


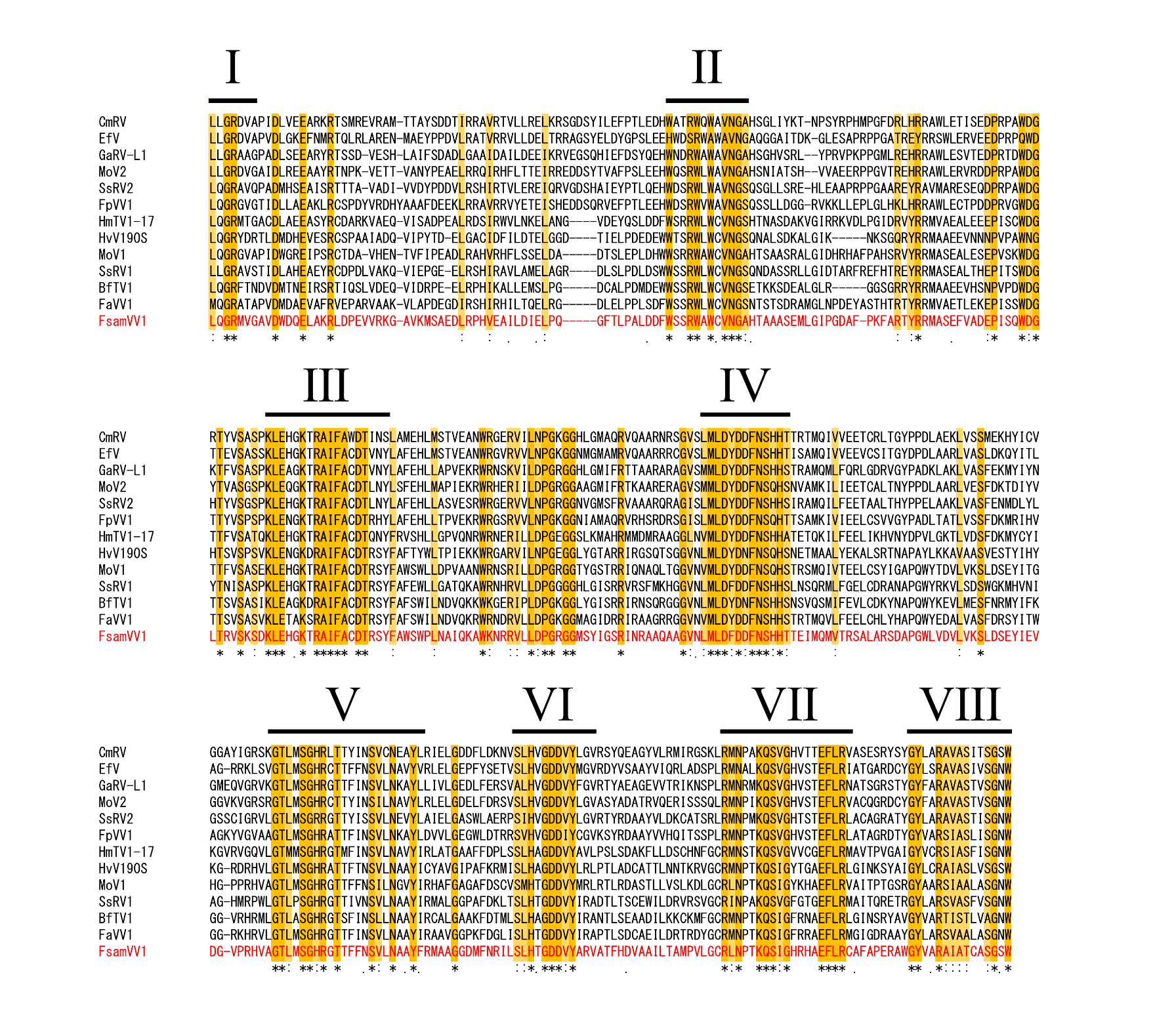


**Supplementary Figure S3.** Amino acid sequence alignment of the RdRp domain of FsamVV1 and selected members of the genus *Victorivirus*. The position of eight core RdRp motifs and conserved residues were numbered and highlighted.

**
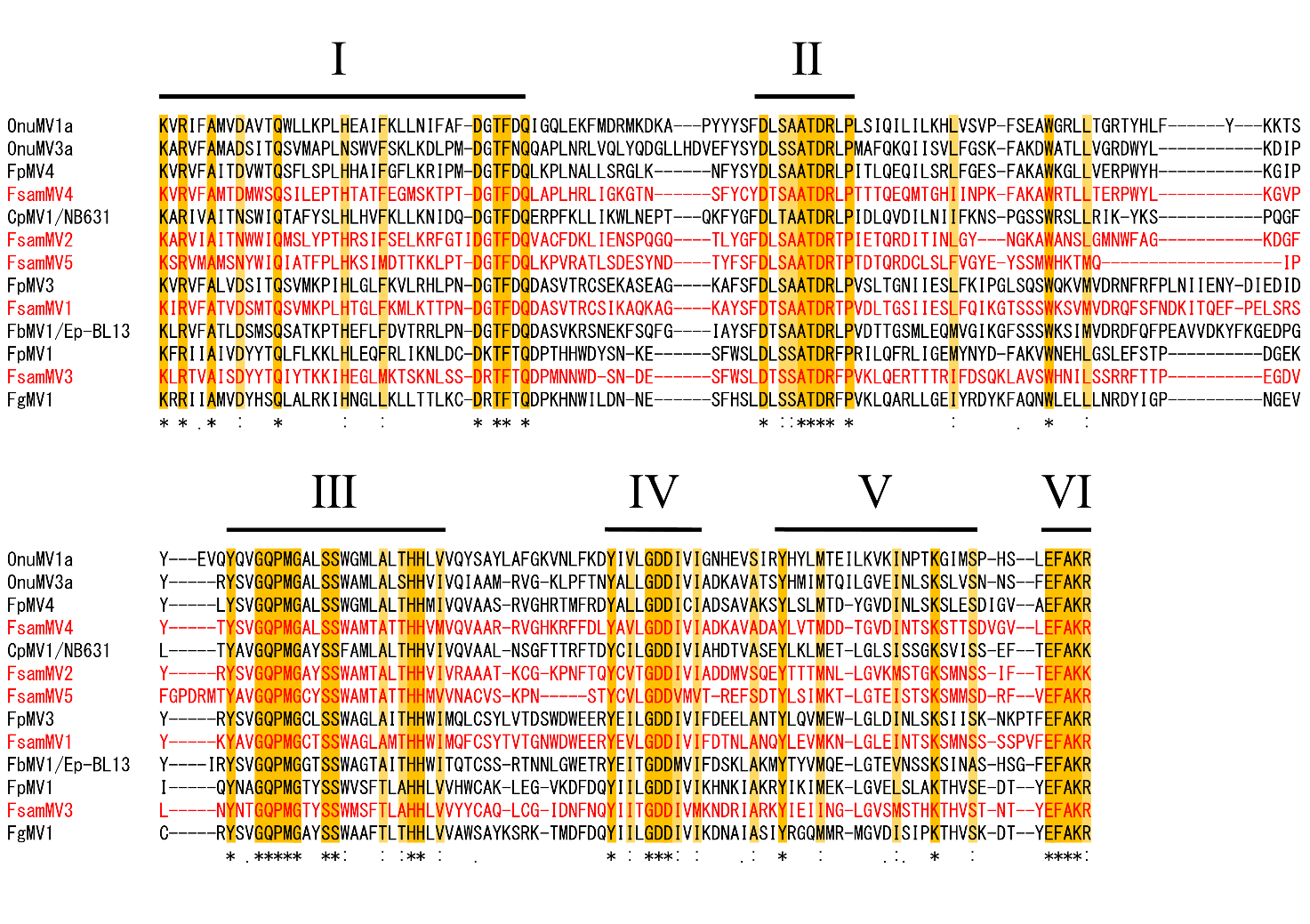
**

**Supplementary Figure S4.** Amino acid sequence alignment of the RdRp domains of FsamMVs and selected members of the genus *Mitovirus*. The position of six core RdRp motifs and conserved residues were numbered and highlighted.


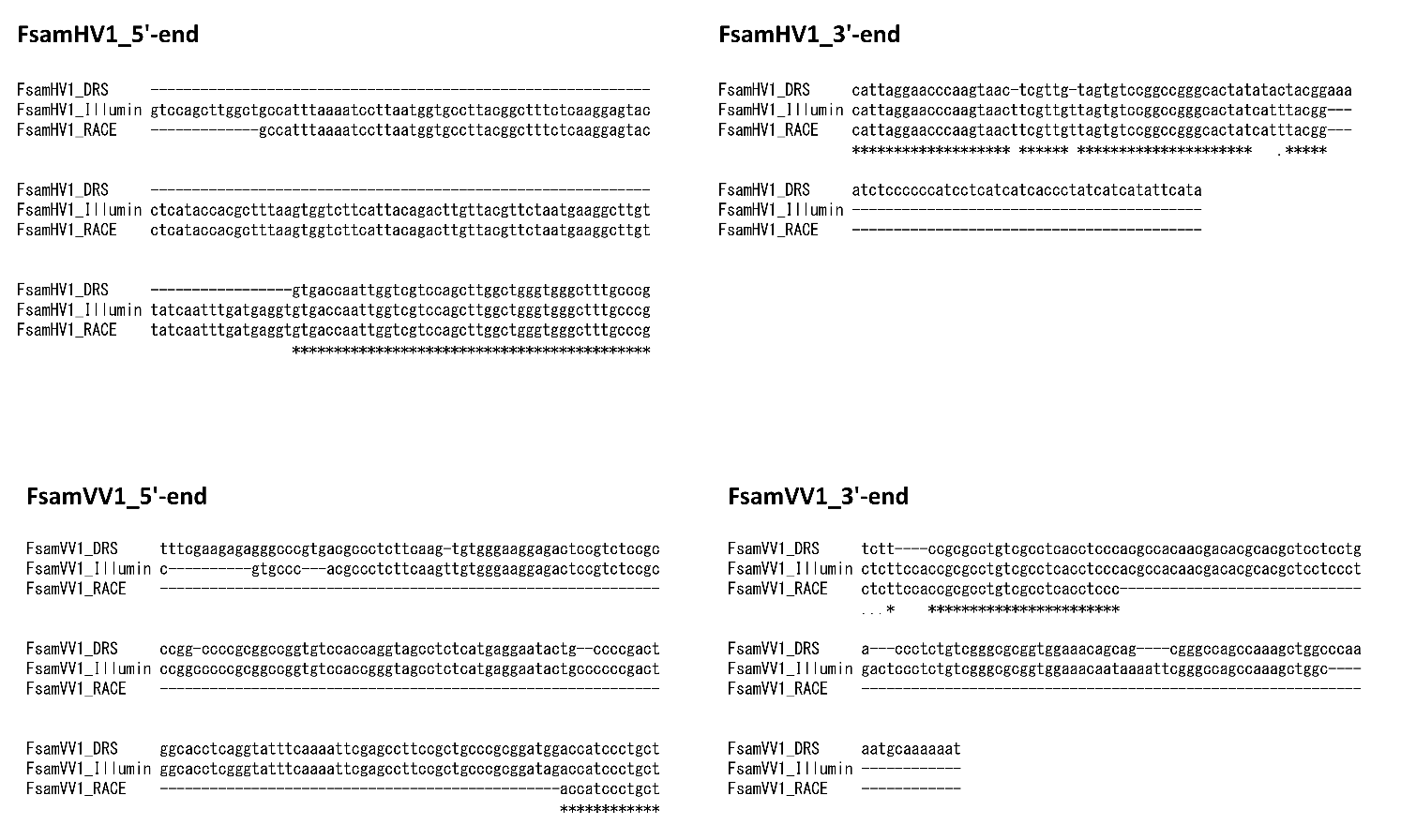


**Supplementary Figure S5.** Nucleotide sequence alignment of the 5’- and 3’-termini of DRS and illumina contigs, and complete genomes of FsamHV1 (Upper) and FsamVV1 (Lower).


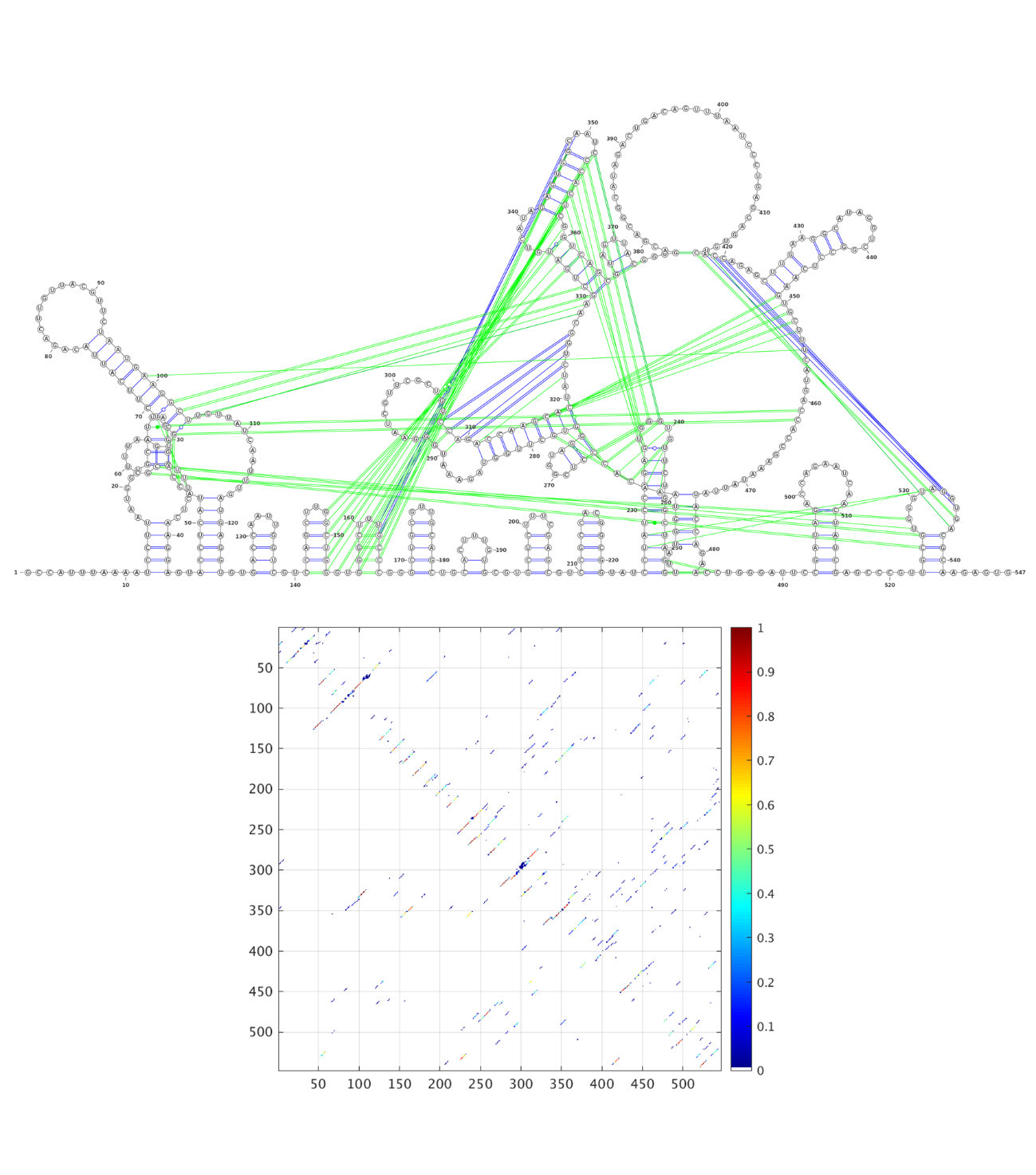


A

B

**Supplementary figure S6.** Predicted RNA secondary structure of FsamHV1’s 5'-UTR sequence. Structure prediction was conducted using SPOT-RNA (https://sparks-lab.org/server/spot-rna/). **(A)** The two-dimensional predicted layout with canonical base-pair (BP) in blue, and non-canonical, lone-pairs and triplets in green. **(B)** Base-pair probability plot predicted by SPOT-RNA (Ensemble of 5 Models) in upper triangle and base-pair probability plot predicted by baseline model of SPOT-RNA (Model 0) in lower triangle.
